# The Immunomodulatory Effects of Albumin *In Vitro* and *In Vivo*

**DOI:** 10.1155/2011/691928

**Published:** 2011-04-26

**Authors:** Derek S. Wheeler, John S. Giuliano, Patrick M. Lahni, Alvin Denenberg, Hector R. Wong, Basilia Zingarelli

**Affiliations:** ^1^Division of Critical Care Medicine, The Kindervelt Laboratory for Critical Care Medicine Research, Cincinnati Children's Research Foundation, Cincinnati Children's Hospital Medical Center, Cincinnati, OH 45229-3039, USA; ^2^Department of Pediatrics, University of Cincinnati College of Medicine, Cincinnati, OH 45229-3039, USA

## Abstract

Albumin appears to have proinflammatory effects *in vitro*. We hypothesized that albumin would induce a state of tolerance to subsequent administration of lipopolysaccharide (LPS) *in vitro* and *in vivo*. RAW264.7 and primary peritoneal macrophages were treated with increasing doses of bovine serum albumin (BSA) and harvested for NF-**κ**B luciferase reporter assay or TNF-**α** ELISA. In separate experiments, RAW264.7 cells were preconditioned with 1 mg/mL BSA for 18 h prior to LPS (10 **μ**g/mL) treatment and harvested for NF-**κ**B luciferase reporter assay or TNF-**α** ELISA. Finally, C57Bl/6 mice were preconditioned with albumin via intraperitoneal administration 18 h prior to a lethal dose of LPS (60 mg/kg body wt). Blood was collected at 6 h after LPS administration for TNF-**α** ELISA. Albumin produced a dose-dependent and TLR-4-dependent increase in NF-**κ**B activation and TNF-**α** gene expression *in vitro*. Albumin preconditioning abrogated the LPS-mediated increase in NF-**κ**B activation and TNF-**α** gene expression *in vitro* and *in vivo*. The clinical significance of these findings remains to be elucidated.

## 1. Introduction

Intravenous fluid resuscitation was first described by Thomas Latta in 1832 [1]. Since that time, early and aggressive intravenous fluid resuscitation has become one of the top management priorities in critically ill children and adults with shock. However, the optimal type of resuscitation fluid, crystalloid or colloid, remains controversial [2–5]. For example, a systematic review of 26 randomized trials involving over 1,500 critically ill patients suggested that the use of albumin for fluid resuscitation increased the absolute risk of mortality by 4% (95% confidence interval 0% to 8%) [3]. A follow-up systematic review suggested that, while there were no differences in mortality or length of hospital stay between isotonic crystalloid and colloid resuscitation, a subgroup analysis suggested that crystalloid resuscitation in critically ill patients with trauma was associated with a lower mortality [4]. A large, multicenter, prospective, randomized trial (SAFE Study) involving nearly 7,000 patients showed that there were no differences in organ failures, intensive care unit (ICU) length of stay, the number of days on mechanical ventilatory support, or mortality between patients assigned to 4% albumin versus normal saline for fluid resuscitation [6]. However, a post hoc study suggested that fluid resuscitation with albumin was associated with higher mortality in patients with traumatic brain injury [7]. Finally, albumin administration was associated with increased mortality and length of stay in a large, European multicenter, observational cohort study performed in over 3,000 critically ill adults [8]. The choice of intravenous fluid may therefore impact outcome in critically ill patients depending upon the particular clinical context. 

The effects of various intravenous fluid preparations on the host inflammatory response are well described [9, 10]. The conflicting data on albumin administration in critically ill patients discussed above support the observation that intravenous fluid preparations are not completely innocuous and may, in fact, potentiate or modulate cellular injury, again depending upon the clinical context. Several studies suggest that albumin circulating in the blood at physiologic concentrations facilitates the interactions between endotoxin- and lipopolysaccharide-binding protein (LBP) and CD-14, thereby assisting with pathogen recognition and the subsequent host inflammatory response [11]. Recent experimental data suggest that exogenous albumin treatment produces a dose-dependent increase in proinflammatory gene expression *in vitro*, primarily through a mechanism involving activation of the transcription factor, NF-*κ*B [12–17]. Albumin may act via the mitogen-activated protein kinase (MAPK) pathway, in this regard [18, 19]. We therefore hypothesized that albumin treatment would modulate the proinflammatory response to LPS* in vitro* and *in vivo*.

## 2. Materials and Methods

### 2.1. Cell Culture

RAW264.7 murine peritoneal macrophages (American Type Culture Collection, Bethesda, Md) were maintained in Dulbecco's Modified Eagle's Medium (DMEM, Gibco BRL, Grand Island, NY) containing 10% fetal bovine serum (FBS), 100 U/mL penicillin, 0.1 mg/mL streptomycin, 20 mM HEPES buffer, and 2.2 g/L sodium bicarbonate (Sigma, St. Louis, Mo) at 37°C in a room air/5% CO_2_ tissue culture incubator. The human acute monocytic leukemia cell line, THP-1, was maintained in RPMI 1640 medium containing 10% FBS, kanamycin, 2-*β*-mercaptoethanol, and 2% glutamine (pH 7.35) at 37°C in a room air/5% CO_2_ tissue culture incubator. Cells were used between passages 3–5.

### 2.2. Isolation of Murine Peritoneal Macrophages

In separate experiments, primary peritoneal macrophages were isolated from C3H/HeJ and C3H/HeOuJ mice (Jackson Laboratory, Bar Harbor, Me) via peritoneal lavage. C3H/HeJ and C3H/HeOuJ mice were housed in a laminar hood in a virus-free animal facility prior to isolation of macrophages. All experiments were conducted in accordance with the National Institutes of Health Guidelines for the Use of Laboratory Animals (National Institutes of Health Publication 85-23, revised 1996) and with approval of the Institutional Animal Care and Use Committee, Cincinnati Children's Research Foundation. Animals were acclimatized for 7 days prior to surgical manipulation and maintained on 12-h light/dark cycles with access to food and water ad libitum. Briefly, mice were anesthetized with isoflurane, and the peritoneal fascia was exposed by dissection. Three mL of sterile PBS was injected through the fascia into the peritoneal cavity using a sterile 22-G needle. Peritoneal fluid was then withdrawn through the fascia with an 18-G needle. The recovered peritoneal fluid was centrifuged at 1500 rpm × 10 min, and the pellet was isolated, resuspended in DMEM containing 10% FBS, and plated on a 96 well plate. Peritoneal macrophages were allowed to adhere for 1 hour at 37°C. Cytospin of the sample was performed to confirm the percentage of macrophages present, which was consistently greater than 90% of the cells in the sample.

### 2.3. Transient Transfection and Luciferase Reporter Assay

RAW264.7 cells were transiently transfected with a 3xNF-*κ*B luciferase reporter plasmid in duplicate, in six-well plates, at a density of 200,000 cells per well by incubation with FuGENE 6 (Roche Molecular Biochemicals, Indianapolis, Ind) and serum-free DMEM overnight. After transfection, cells were washed once with PBS and treated with increasing concentrations of bovine serum albumin (BSA) (Sigma-Aldrich, St. Louis, Mo). In separate experiments, cells were preconditioned with BSA × 18 h at 37°C prior to subsequent treatment with LPS, 10 *μ*g/mL × 6 h at 37°C. 

In separate experiments, THP-1 cells were transfected using DEAE-dextran. Briefly, 1 × 10^6^ THP-1 cells/mL were seeded into tissue culture flasks the day before transfection. On the next day, 6 mL of cell suspension were washed twice with STBS (25 mM Tris · Cl, pH 7.4, 137 mM NaCl, 5 mM KCl, 0.6 mM Na_2_HPO_4_, 0.7 mM CaCl_2_, and 0.5 mM MgCl_2_) and pelleted. One *μ*g/mL of NF-*κ*B reporter plasmid was mixed with DEAE-dextran (400 *μ*g/mL) in 140 *μ*L of STBS buffer and immediately added to the pelleted THP-1 cells. The cells were incubated at 37°C for 20 min, washed twice with STBS, resuspended, and cultured in complete RPMI medium. The transfected cell lines were cultivated for 48 h and harvested. 

Cellular proteins were extracted and analyzed for luciferase activity according to the manufacturer's instructions (Promega) using a Berthold AutoLumat LB953 luminometer. Luciferase activity was corrected for total cellular protein and reported as fold induction over control cells (cells that were transfected and treated with medium alone).

### 2.4. Enzyme-Linked Immunosorbent Assay (ELISA)

RAW264.7 cells or primary peritoneal macrophages were preconditioned with BSA, 1 mg/mL × 18 h at 37°C prior to subsequent treatment with LPS, 10 *μ*g/mL × 6 h at 37°C. In separate experiments, male C57Bl/6 mice were preconditioned *in vivo* with BSA, 10 *μ*g/kg body wt × 18 h. Primary peritoneal macrophages were then isolated, allowed to adhere, and treated *ex vivo* with LPS, 10 *μ*g/mL × 6 h at 37°C. Cell supernatants were collected and clarified (5,000 rpm for 10 min at 4°C) prior to being analyzed for tumor necrosis factor- (TNF-) *α* via ELISA (BioSource International, Camarillo, Calif) using the protocol recommended by the manufacturer.

### 2.5. Nuclear Protein Extraction and EMSA

Nuclear proteins were isolated from treated cells as previously described [20] and stored at −70°C until further analysis. EMSA was performed using an oligonucleotide probe for the NF-*κ*B consensus site as previously described [20].

### 2.6. Murine Model of Endotoxin Shock

C57Bl/6 mice (Jackson Laboratory, Bar Harbor, Me), 20–25 g body weight, were acclimatized for 7 days prior to surgical manipulation and maintained on 12-h light/dark cycles with access to food and water ad libitum. Briefly, mice were preconditioned with bovine serum albumin (Sigma-Aldrich, St. Louis, Mo), 10 mg/kg body wt or vehicle, administered via intraperitoneal (i.p.) injection and were returned to their cages. After 18 h, 60 mg/kg body weight of lipopolysaccharide (LPS) (Sigma-Aldrich, St. Louis, Mo) was administered via i.p. injection. Mice were anesthetized 6 hours later with isoflurane, and blood was obtained via direct cardiac puncture and centrifuged at 5,000 g × 10 minutes in EDTA-calcium vacutainer. The plasma was then collected and stored at −80°C until further analysis. Plasma concentration of tumor necrosis factor- (TNF-) *α* was measured via commercially available enzyme-linked immunosorbent assay kits (Biosource International, Camarillo, Calif) using the protocol recommended by the manufacturer.

### 2.7. Statistical Analysis

All continuous data are reported as mean ± SEM and were compared using one-way analysis of variance and Student Newman-Keuls test (Stata 11.1, StataCorp, College Station, Tex). A *P* value <.05 was considered statistically significant.

## 3. Results

### 3.1. Albumin Dose-Dependently Induces NF-*κ*B-Dependent, TLR-4-Dependent TNF-*α* Gene Expression in Murine Peritoneal Macrophages

**Figure 1:**
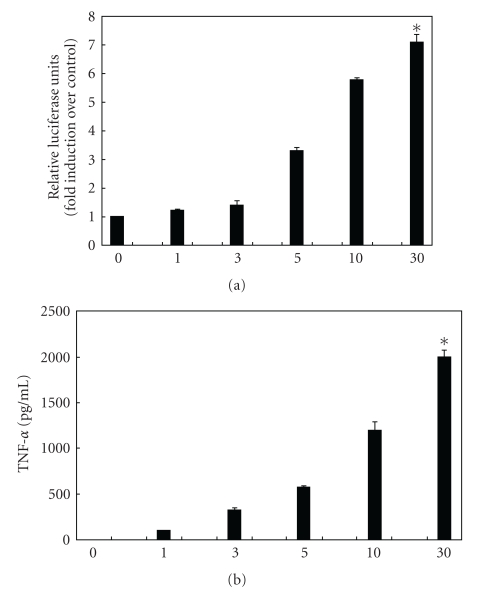
Treatment with fraction V bovine serum albumin (BSA) (Sigma-Aldrich, St. Louis, Mo) dose-dependently increases TNF-*α* gene expression in murine peritoneal macrophages in an NF-*κ*B-dependent manner. (a) RAW264.7 peritoneal macrophages were transiently transfected with a 3x NF-*κ*B/luc reporter plasmid prior to treatment with increasing doses of BSA for 24 h (doses in mg/mL shown on the *x*-axis). BSA treatment resulted in increased NF-*κ*B activation in a dose-dependent manner. As a comparison, LPS treatment (10 *μ*g/mL) resulted in a 3.9-fold increase in relative luciferase activity (*data not shown*). All experiments were performed in triplicate with 3 wells per condition (*P < .05 compared to control). (b) RAW264.7 peritoneal macrophages were treated with increasing doses of BSA for 24 h (doses in mg/mL shown on the *x*-axis). BSA treatment resulted in a significant increase in TNF-*α*, as measured by ELISA. As a comparison, LPS treatment (10 *μ*g/mL) resulted in a much greater increase in TNF-*α* compared to BSA (TNF-*α* 7000 pg/mL) (*data not shown*). All experiments were performed in triplicate with 3 wells per condition (*P < .05 compared to control).

Prior studies have shown that exogenous albumin treatment produces a dose-dependent increase in proinflammatory gene expression *in vitro*, primarily through a mechanism involving activation of the transcription factor, NF-*κ*B [12–16]. In order to confirm these results, we transfected muirine peritoneal macrophages (RAW264.7) with a 3x-NF-*κ*B luciferase reporter plasmid prior to treatment with increasing doses (0.5–30 mg/mL) of Fraction V BSA (Sigma-Aldrich, St. Louis, Mo). BSA treatment resulted in a dose-dependent increase in NF-*κ*B promoter activity. Similarly, BSA treatment resulted in a dose-dependent increase in the NF-*κ*B-dependent proinflammatory cytokine, TNF-*α*, as measured by ELISA (Figure 1).

In order to confirm that these results were not due to potential endotoxin contamination of the commercial albumin preparation, we repeated the experimental conditions in the presence of the endotoxin inhibitor, polymyxin B (100 *μ*g/mL added 1 h before albumin or LPS treatment, resp.). While polymyxin B reduced BSA-induced TNF-*α* expression, the difference was not statistically significant (BSA 30 mg/mL: 1995 ± 75 pg/mL versus BSA 30 mg/mL + polyB: 1100 ± 50 pg/mL, *P* = NS). In stark contrast, polymyxin B cotreatment significantly inhibited LPS-mediated TNF-*α* expression back to baseline (LPS 10 *μ*g/mL: 7,000 ± 200 pg/mL versus LPS 10 *μ*g/mL + polyB: 50 ± 0 pg/mL, *P* < .05). In addition, we repeated the ELISA experiments using a low-endotoxin albumin preparation (Sigma-Aldrich, St. Louis, Mo). Both albumin preparations significantly increased TNF-*α* expression compared to control, and there were no significant differences between Fraction V BSA versus low-endotoxin albumin (*data not shown*). Finally, as an additional control, we independently verified the level of endotoxin contamination in our albumin preparation via Limulus amoebocyte lysate (LAL) assay performed at Charles River Laboratories (Charleston, SC). The level of endotoxin contamination (0.8828 EU/mL) at the highest albumin dose studied (30 mg/mL) was equivalent to a LPS dose of 12 pg/mL. We treated RAW264.7 transfected with 3x NF-*κ*B luciferase reporter plasmid with 12 pg/mL LPS, which was not significantly different from control cells (*data not shown*). Collectively, these studies convincingly show that exogenous BSA induces proinflammatory gene expression independent of any potential endotoxin contamination.

As an additional control to show that these effects were not due to species-dependent differences (i.e., treating mouse peritoneal macrophages with a bovine preparation of albumin), we treated THP-1 cells transfected with the 3x NF -*κ*B luciferase reporter plasmid with human recombinant albumin (Sigma-Aldrich, St. Louis, Mo). Human albumin dose-dependently increased NF-*κ*B activation in the human THP-1 cell line (Alb 1 mg/mL: 1.5-fold induction; Alb 3 mg/mL: 5.0-fold induction; Alb 10 mg/mL: 6.5 fold induction; Alb 30 mg/mL: 6.0-fold induction; *P* < .05).

We wanted to confirm that these findings were not just a nonspecific effect of a high concentration of a high-molecular-weight protein. We next treated RAW264.7 cells transfected with the 3x NF-*κ*B luciferase reporter plasmid with increasing doses of the high-molecular-weight protein, Dextran (Sigma-Aldrich, St. Louis, Mo). In contrast to the results observed with BSA above, Dextran treatment did not increase NF-*κ*B activation, even at the highest dose studied (30 mg/mL) (*data not shown*). 

**Figure 2:**
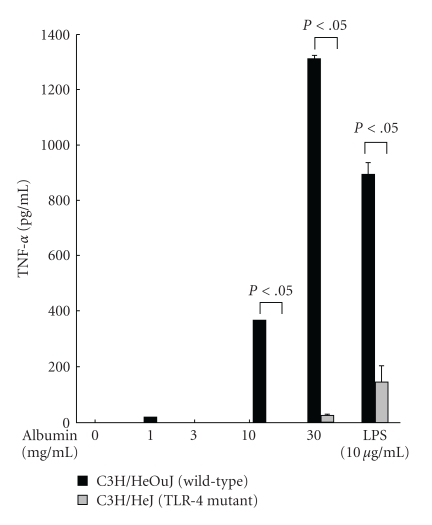
BSA increases TNF-*α* gene expression in murine peritoneal macrophages in a TLR-4-dependent manner. Primary peritoneal macrophages were isolated from C3H/HeJ (TLR-4 mutant) and C3H/HeOuJ (wild-type) and were treated with BSA for 24 h. As expected, LPS treatment (10 *μ*g/mL) resulted in a significant difference in TNF-*α* induction between macrophages isolated from C3H/HeJ mice and their wild-type counterparts. BSA treatment resulted in a dose-dependent increase in TNF-*α* in macrophages isolated from C3H/HeOuJ mice, but not in macrophages isolated from C3H/HeJ mice. All experiments were performed in triplicate with 3 wells per condition.

Finally, previous studies suggest that exogenous albumin does not induce proinflammatory gene expression in macrophages [17]. We therefore wished to confirm our results in primary murine macrophages. We also wanted to determine whether the proinflammatory effects of exogenous albumin administration were dependent upon the Toll-like receptor- (TLR-) 4 pathway [20, 21]. We therefore treated peritoneal macrophages obtained from both LPS-resistant (due to a mutation in the TLR-4 receptor) C3H/HeJ mice and LPS-sensitive C3H/HeOuJ mice. BSA treatment resulted in a dose-dependent increase in TNF-*α*, as measured by ELISA, in peritoneal macrophages of C3H/HeOuJ mice only (Figure 2). Collectively, these data extend the findings from previous studies [12–16] and further demonstrate that exogenous albumin treatment produces a dose-dependent increase in proinflammatory gene expression in a NF-*κ*B-dependent manner. Moreover, exogenous albumin produced a dose-dependent increase in TNF-*α* in peritoneal macrophages obtained from LPS-sensitive C3H/HeOuJ mice, but not in LPS-resistant C3H/HeJ mice, suggesting that these effects are dependent upon the Toll-like receptor- (TLR-) 4 pathway.

### 3.2. Albumin Preconditioning Abrogates LPS-Mediated TNF-*α* Gene Expression in RAW264.7 Macrophages

**Figure 3:**
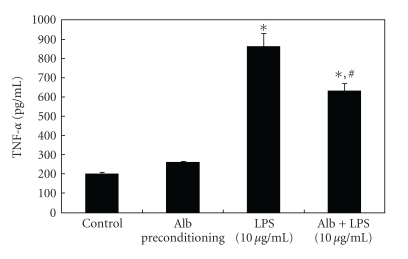
Albumin preconditioning abrogates LPS-mediated TNF-*α* gene expression in RAW264.7 macrophages. RAW264.7 peritoneal macrophages were preconditioned with BSA (1 mg/mL) for 18 h prior to a subsequent treatment with LPS (10 *μ*g/mL). LPS treatment resulted in a significant increase in TNF-*α* expression, as measured by ELISA. Albumin preconditioning, however, significantly abrogated LPS-mediated TNF-*α* expression. All experiments were performed in triplicate with 3 wells per condition (*P < .05 compared to control; ^#^ *P* < .05 compared to LPS alone).

**Figure 4:**
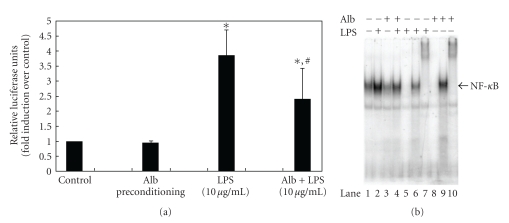
Albumin preconditioning attenuates LPS-mediated NF-*κ*B activation. (a) NF-*κ*B luciferase experiments. RAW264.7 peritoneal macrophages were transiently transfected with a 3x NF -*κ*B/luc reporter plasmid. Cells were allowed to recover overnight and were then preconditioned with BSA (1 mg/mL) for 18 h prior to a subsequent treatment with LPS (10 *μ*g/mL). LPS treatment resulted in a significant increase in NF-*κ*B promoter activation, which was significantly inhibited by albumin preconditioning. All experiments were performed in triplicate with 3 wells per condition (*P < .05 compared to control; ^#^ *P* < .05 compared to LPS alone). (b) EMSA. RAW264.7 peritoneal macrophages were preconditioned with BSA (1 mg/mL) for 18 h prior to a subsequent treatment with LPS (10 *μ*g/mL). Nuclear protein was harvested at 30 min after LPS and EMSA were performed. LPS treatment resulted in a significant increase in NF-*κ*B binding (Lane 2) compared to either control (Lane 1) or albumin preconditioning alone (Lane 3). Albumin preconditioning abrogated NF-*κ*B binding (Lane 4) compared to LPS alone. Lane 5 (LPS treatment, cold competitor), Lane 6 (LPS treatment, p65 supershift), Lane 7 (LPS treatment, p50 supershift), Lane 8 (Alb + LPS, cold competitor), Lane 9 (Alb + LPS, p65 supershift), and Lane 10 (Alb + LPS, p50 supershift) were performed as additional controls to demonstrate specificity and the nature of the NF-*κ*B. The EMSA shown is representative of 3 separate experiments, all with similar results.

Given the effects of albumin on TNF-*α* expression *in vitro *and the potential role of TLR-4 in this process, we hypothesized that albumin preconditioning would attenuate subsequent LPS-mediated TNF-*α* expression in RAW264.7 macrophages, similar to the phenomenon of endotoxin tolerance [20, 21]. We therefore preconditioned RAW264.7 macrophages with a low dose (i.e., a dose that did not significantly activate NF-*κ*B activity or TNF-*α* expression) of BSA, 1 mg/mL × 18 h at 37°C, prior to a subsequent treatment with LPS, 10 *μ*g/mL. As shown in Figure 3, albumin preconditioning abrogated LPS-mediated TNF-*α* expression. We next transfected RAW264.7 cells with the 3x-NF-*κ*B luciferase reporter plasmid and repeated these experiments. As expected, LPS treatment resulted in a significant increase in NF-*κ*B promoter activation. Albumin preconditioning significantly inhibited subsequent LPS-mediated NF-*κ*B promoter activation (Figure 4(a)). These results were further confirmed by EMSA (Figure 4(b)).

We wanted to confirm that these findings were not just a nonspecific effect of a high concentration of a high-molecular-weight protein. We therefore preconditioned RAW264.7 cells transfected with the 3x NF -*κ*B luciferase reporter plasmid with increasing doses of the high-molecular weight protein Dextran (1–30 mg/mL) prior to subsequent LPS treatment. Again, in contrast to the results observed with BSA preconditioning, Dextran preconditioning did not inhibit LPS-mediated NF-*κ*B activation (LPS treatment: 3.9-fold induction versus Dextran + LPS: 4.1-fold induction, *P* = NS). Collectively, these data confirm that albumin preconditioning attenuates LPS-mediated TNF-*α* gene expression and NF-*κ*B activation *in vitro*.

### 3.3. Albumin Abrogates LPS-Mediated TNF-*α* Gene Expression *In Vivo*

**Figure 5:**
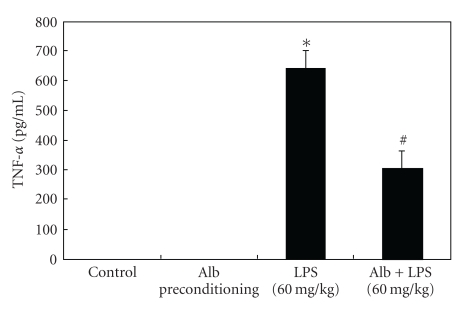
Albumin preconditioning abrogates LPS-mediated TNF-*α* expression* in vivo*. C57Bl/6 mice, 20–25 g body wt, were preconditioned with BSA (10 mg/kg body wt, i.p.) or vehicle 18 h prior to subsequent treatment with a lethal dose of LPS (60 mg/kg body wt, i.p.). Plasma was harvested at 6 h, and TNF-*α* was measured via ELISA. Albumin preconditioning significantly inhibited LPS-mediated plasma TNF-*α* expression. Experiments were performed in triplicate with 5 mice per experimental group (*P < .05 compared to control; ^#^ *P* < .05 compared to LPS alone).

Albumin is one of the most abundant serum proteins *in vivo*. Given the immunomodulatory effects of physiologic levels of albumin [11], it is difficult to discern whether the immunomodulatory effects observed in the *in vitro* experiments above would be observed following the administration of exogenous albumin in the clinical setting. For this reason, we wanted to confirm that exogenous albumin treatment could abrogate LPS-mediated TNF-*α* gene expression *in vivo*. Our group has previously shown that intraperitoneal administration of LPS, 60 mg/kg results in significant induction of TNF-*α* at 6 h after injection [22]. Six-week-old C57Bl/6 mice (20–25 g) were preconditioned with either saline vehicle or BSA (10 mg/kg i.p.) 18 h prior to a subsequent challenge with LPS, 60 mg/kg. Consistent with our previous study [22], LPS injection produced a significant increase in plasma TNF-*α*, which was significantly abrogated by albumin preconditioning (Figure 5). Collectively, these data demonstrate that albumin preconditioning modulates the proinflammatory response to LPS *in vivo*, suggesting that administration of exogenous albumin may have immunomodulatory effects in the clinical setting.

## 4. Discussion

Herein, we confirm previous *in vitro* studies which demonstrate that albumin increases proinflammatory gene expression in an NF-*κ*B-dependent manner [12–14, 16]. We extend these findings to show that albumin exerts these proinflammatory effects through Toll-like receptor- (TLR-) 4. Finally, we demonstrate that albumin pretreatment, or preconditioning, significantly attenuates subsequent LPS-mediated TNF-*α* gene expression *in vitro* and *in vivo*. Albumin preconditioning therefore elicits a cellular response similar to classic endotoxin tolerance [21, 23]. For example, while LPS induces a dramatic increase in TNF-*α* gene expression in human peripheral blood monocytes (PBMCs), a second exposure produces a markedly attenuated response with decreased TNF-*α* gene expression [24]. The common assertion that endotoxin tolerance represents a global downregulation of proinflammatory gene expression, however, is perhaps incomplete and not entirely accurate. Whereas TNF-*α* production is significantly diminished, production of other proinflammatory cytokines such as IL-1*β* and IL-6 may be increased, decreased, or unchanged [23]. In addition, early studies suggested that endotoxin tolerance resulted in both (i) a diminished proinflammatory response to a subsequent dose of LPS *in vitro* and *in vivo* and (ii) *increased* phagocytosis *in vitro *[21]. As such, the clinical relevance of endotoxin tolerance remains unknown. 

Albumin appears to have important immunomodulatory effects that likely impact the host inflammatory response in critical illness. The impact of these immunomodulatory effects may, in turn, depend upon the clinical context (i.e., sepsis, hemorrhagic shock, or trauma). For example, the immunomodulatory effects of albumin in critically ill patients with a predominantly proinflammatory phenotype may improve outcome, whereas these same effects may worsen outcome in critically ill patients with a predominantly anti-inflammatory phenotype. Consistent with this notion, albumin fluid resuscitation prevents experimental lung injury following hemorrhagic shock [25–27], but not after endotoxic shock [26]. These questions remain an active focus of investigation in many laboratories, including our own. 

Several endogenous molecules, including several members of the heat shock protein (Hsp) family of proteins (e.g. Hsp10, Hsp60, and Hsp72) [28–30], ubiquitin [31], uric acid [32], and HMGB-1 [33, 34], have recently been shown to modulate the host inflammatory response *in vitro*. Recent studies have questioned some of these findings due to the possibility of bacterial contamination of the recombinant proteins used in the aforementioned studies [35, 36]. With this in mind, we performed several additional controls in order to assure that endotoxin contamination of our protein preparation was not responsible for these effects. First, we independently verified the level of endotoxin contamination in our protein preparation and showed that this concentration of endotoxin did not induce TNF-*α* gene expression. Next, we treated cells with a commercially available “endotoxin-free” albumin preparation and noted similar results. Finally, we conducted our experiments in the presence of polymyxin B. While polymyxin B reduced the proinflammatory effects of albumin, the difference was not statistically significant. Collectively, these results suggest that albumin has immunomodulatory properties that are distinct from any bacterial contamination of the protein preparation, consistent with previous observations [12, 16].

In conclusion, we show that albumin has potent immunomodulatory effects *in vitro* and *in vivo*. Albumin induces TNF-*α* gene expression in RAW264.7 peritoneal macrophages in a TLR4- and NF-*κ*B-dependent manner. These effects are not due to endotoxin contamination of the recombinant protein. The clinical significance of these effects remains to be elucidated and warrants further investigation.
